# Palmitoylation regulates the magnitude of HCN4-mediated currents in mammalian cells

**DOI:** 10.3389/fphys.2023.1163339

**Published:** 2023-04-13

**Authors:** Samitha Dilini Congreve, Alice Main, Andrew S. Butler, Xing Gao, Elaine Brown, Chunyun Du, Stephanié C. Choisy, Hongwei Cheng, Jules C. Hancox, William Fuller

**Affiliations:** ^1^ School of Cardiovascular & Metabolic Health, University of Glasgow, Glasgow, United Kingdom; ^2^ School of Physiology, Pharmacology and Neuroscience, University of Bristol, Bristol, United Kingdom

**Keywords:** sinoatrial node, funny current, acylation, heart rate, ion channel, palmitoylation

## Abstract

The sinoatrial node (SAN) and subsidiary pacemakers in the cardiac conduction system generate spontaneous electrical activity which is indispensable for electrical and therefore contractile function of the heart. The hyperpolarisation-activated cyclic nucleotide-gated channel HCN4 is responsible for genesis of the pacemaker “funny” current during diastolic depolarisation. S-palmitoylation, the reversible conjugation of the fatty acid palmitate to protein cysteine sulfhydryls, regulates the activity of key cardiac Na^+^ and Ca^2+^ handling proteins, influencing their membrane microdomain localisation and function. We investigated HCN4 palmitoylation and its functional consequences in engineered human embryonic kidney 293T cells as well as endogenous HCN4 in neonatal rat ventricular myocytes. HCN4 was palmitoylated in all experimental systems investigated. We mapped the HCN4 palmitoylation sites to a pair of cysteines in the HCN4 intracellular amino terminus. A double cysteine-to-alanine mutation CC93A/179AA of full length HCN4 caused a ∼67% reduction in palmitoylation in comparison to wild type HCN4. We used whole-cell patch clamp to evaluate HCN4 current (I_HCN4_) in stably transfected 293T cells. Removal of the two N-terminal palmitoylation sites did not significantly alter half maximal activation voltage of I_HCN4_ or the activation slope factor. I_HCN4_ was significantly larger in cells expressing wild type compared to non-palmitoylated HCN4 across a range of voltages. Phylogenetic analysis revealed that although cysteine 93 is widely conserved across all classes of HCN4 vertebrate orthologs, conservation of cysteine 179 is restricted to placental mammals. Collectively, we provide evidence for functional regulation of HCN4 *via* palmitoylation of its amino terminus in vertebrates. We suggest that by recruiting the amino terminus to the bilayer, palmitoylation enhances the magnitude of HCN4-mediated currents, but does not significantly affect the kinetics.

## Introduction

The intrinsic automaticity of the sinoatrial node (SAN) is fundamental for pacemaking in mammalian hearts. In cells of the SAN (and elsewhere in the cardiac conduction system) slow depolarisation during diastole is the basis of this automaticity, distinguishing these specialised cells from the working myocardium (Choudhury et al., 2015). Multiple electrogenic processes contribute to this diastolic depolarisation. Surface membrane ion currents (“membrane clock”) and the rhythmic oscillation of local calcium release (“calcium clock”) work interdependently and are believed to form a coupled-clock system that drives pacemaker automaticity and its regulation on a beat-to-beat basis (DiFrancesco, 2010; Lakatta et al., 2010).

The hyperpolarisation-activated cyclic nucleotide-gated channel HCN4 is a key component of the membrane clock. HCN4 is the predominant HCN isoform expressed in the sinoatrial node (Bucchi et al., 2012), contributing 75%–90% of the sinoatrial “funny” current (I_f_) during diastolic depolarisation (Stieber et al., 2003; Baruscotti et al., 2011). This native cardiac I_f_ current carried by HCN4 was christened “funny” when it was first identified because of its “funny” (unusual) property of being activated by hyperpolarisation (Brown et al., 1979; DiFrancesco, 2019). HCN channels are tetrameric, with each subunit consisting of 6 transmembrane (TM) domains, with the voltage sensor in TM4, a re-entrant loop between TM5 and 6 forming the channel pore and cytosolic amino and carboxyl termini (Saponaro et al., 2021). Unique properties of HCN channels such as the depolarising mixed (Na^+^ and K^+^) inward current on membrane hyperpolarisation over the diastolic range of voltages, facilitate the channel’s contribution to the early diastolic depolarisation (DiFrancesco, 2010). In addition, a cyclic nucleotide binding site in the carboxyl terminus enables direct activation of HCN channels by cAMP binding without the classic effector kinase (Baruscotti et al., 2005). Loss of function mutations of HCN4 are associated with sinus node dysfunction (Verkerk and Wilders, 2015). Tamoxifen-induced HCN4 knockout in the adult murine heart produces profound bradycardia and also atrioventricular (AV) block, indicating roles for HCN4 not just in the SAN but also in the AV node (Baruscotti et al., 2011). Ivabradine, the first clinically approved drug that selectively inhibits the funny current in the SAN, is used therapeutically to slow the heart rate specifically in the settings of heart failure and chronic stable angina (DiFrancesco and Camm, 2004).

Regulation of ion channels *via* post translational modifications is an integral component of the complex sinoatrial pacemaking network (Tsutsui et al., 2018). For example, phosphorylation of key calcium handling proteins that constitute the calcium clock accelerates diastolic depolarisation and increases heart rate (Lakatta et al., 2010). S-palmitoylation is a form of lipidation that involves the covalent addition of a 16-carbon palmitate to a thiol group of a cysteine residue in a protein (Linder and Deschenes, 2007). Palmitoylation is uniquely reversible amongst protein lipid modifications. A zDHHC-motif containing family of integral membrane palmitoyl acyl transferase (zDHHC-PATs) palmitoylates proteins (Rana et al., 2018), and this reaction is reversed by thioesterases (Won, 2018). Hence palmitoylation facilitates the dynamic regulation of both soluble and integral membrane proteins (Guan and Fierke, 2011; Main and Fuller, 2022).

In recent years, palmitoylation has emerged as an important post translational modification regulating many physiological and pathophysiological processes in the heart (Essandoh et al., 2020; Main et al., 2022; Main and Fuller, 2022). Essential cardiac sodium (Reilly et al., 2015; Pei et al., 2016; Gok et al., 2020; Gok et al., 2022) and calcium (Kuo et al., 2023) handling proteins are dynamically palmitoylated, influencing their membrane microdomain localisation and function. Palmitoylation regulates the activities of several proteins known to be central to SAN pacemaking, including NCX1 (Reilly et al., 2015; Gok et al., 2020) and Ca(v)1.2,^25^ and all isoforms of HCN channels except HCN3 are palmitoylated (Itoh et al., 2016). However, functional regulation of HCN4 channels by palmitoylation has not yet been established. The present *in-vitro* study was undertaken to characterise palmitoylation of HCN4 channels and to establish its functional consequences. The findings demonstrate that HCN4 is primarily palmitoylated at its N-terminus at cysteine 93 and 179. Mutations of both cysteines significantly reduced palmitoylation of HCN4 and altered the magnitude but not activation kinetics of macroscopic HCN4 current.

## Methods

### Ethics statement

This study utilized cardiac tissue from rat and rabbits. All protocols involving animals were approved by the University of Glasgow or University of Bristol Animal Welfare and Ethics Review Board. Rodent cardiac tissues were collected post-mortem after sacrificing animals using a method designated Schedule 1 by the Animals (Scientific Procedures) Act 1986. Rabbit hearts were excised following humane killing in accordance with UK Home Office legislation.

### Mutagenesis and cloning

Cysteine-to-alanine substitution reactions were performed using Q5-site directed mutagenesis kit (New England Biolabs). The primers for the mutagenesis reactions were designed using the manufacturer’s online primer design tool.

The InFusion cloning system was used to subclone full length HCN4. The amino (1-266) terminus and the carboxyl (518-1203) terminus were amplified from the human full length HCN4 in pcDNA 3 and inserted into pEYFP-C1 (Clontech) to express as fusions to the YFP C-terminus. Full-length HCN4 was amplified and inserted in pcDNA5/FRT/TO (Invitrogen).

### Cells and tissue

Cell lines stably expressing tetracycline-inducible wild type and mutant HCN4 channels were generated using the Flp-In™ T-Rex™ System. All transient transfections of plasmid DNA were carried out in a 6 or 12 multi-well plate seeded with a high cell density using Invitrogen Lipofectamine 2000 as instructed by the manufacturer. The cells were harvested 18–24 h following transfection.

Neonatal rat whole hearts were obtained from male and female Sprague Dawley rats aged 1–4 days old. Briefly, animals were euthanised with a lethal dose of Euthatal before severing the femoral artery. Hearts were quickly excised and placed into a solution of ice-cold excision buffer (100 mM NaCl, 20 mM HEPES, 0.8 mM NaH_2_PO_4_, 5.3mM KCl, 0.4 mM MgSO_4_, 5 mM glucose; pH 7.4). Excess blood and tissue were removed from hearts using a sterile scalpel. For analysis of atrial tissue only, the atria were identified and separated from the ventricular tissue.

SAN tissue was obtained from adult male New Zealand White rabbits. The heart was rapidly excised and cleared of blood. The SAN region in the right atrium was identified in relation to known landmarks (the crista terminalis, superior vena cava, interatrial septum and inferior vena cava (Denyer and Brown, 1990; Brioschi et al., 2009), and tissue encompassing the entire SAN region was excised and snap-frozen for subsequent determination of HCN4 palmitoylation.

### Palmitoylation assays

Palmitoylated proteins were purified using resin-assisted capture of acylated proteins (acyl-RAC) (Forrester et al., 2011). Briefly, cells were lysed in blocking buffer (2.5% SDS, 1 mM EDTA, 100 mM HEPES, 1% v/v MMTS pH 7.5) and incubated at 40°C for 4 h to alkylate free cysteines. Proteins were precipitated with 3 volumes of ice-cold acetone to remove excess unreacted MMTS, protein pellets extensively washed with 70% acetone, dried, and redissolved in binding buffer (1% SDS, 1 mM EDTA, 100 mM HEPES pH 7.5). Palmitoylated proteins were captured by agitating for 2.5 h with thiopropyl sepharose beads in the presence of 250 mM neutral hydroxylamine sulphate. In some reactions hydroxylamine was replaced with sodium chloride as a negative control. After capture, beads were extensively washed with binding buffer, and palmitoylated proteins eluted in SDS PAGE loading buffer supplemented with 100 mM DTT.

Intact cardiac tissue was homogenised using ceramic beads (BeadBug) in blocking buffer for 4 h at 40°C. Any unhomogenised tissue was discarded before the precipitation step.

### Western blotting

This investigation used antibodies raised to HCN4 (1:5000-1:10000 dilution Alomone labs #APC-052), caveolin-3 (1:2000 dilution, BD Biosciences 610420), flotillin-2 (1:2000 dilution, BD Bioscences 610383), GFP (1:5000 dilution, Abcam ab6556) and Na^+^/K^+^ ATPase α1 subunit (1:100 dilution, Developmental Studies Hybridoma Bank clone α6F). Secondary antibodies were anti-rabbit conjugated to HRP (Jackson ImmunoResearch, 111-035-144, raised in goat) and anti-mouse conjugated to HRP (Jackson ImmunoResearch, 315-035-003, raised in rabbit).

Images of western immunoblots were acquired using a BioRad Chemidoc XRS imaging system and analysed using QuantityOne (BioRad) and ImageLab (BioRad) software.

### Preparation of surface membrane proteins

Surface membrane proteins were biotinylated by incubating cells in 1 mg/mL sulfo-NHS-SS-biotin in PBS for 30min at 37°C. Cells were lysed in 1% Triton X-100, 0.1% SDS in PBS supplemented with protease inhibitor cocktail. Biotinylated proteins were captured using streptavidin sepharose (Tulloch et al., 2011). Only surface membrane proteins, not intracellular proteins, are captured using this experimental design (Gao et al., 2022).

### Whole cell patch clamp

Stably transfected Flp-In™ 293 T-REx cells (Thermo) were plated on 10 mm glass coverslips in a 35 mm culture dish and HCN4 expression induced with tetracycline (1 µg/mL) for at least 24 h prior to being used for electrophysiology recordings. Whole cell patch clamp recordings employed an internal solution comprised of (in mmol/L): 130 KCl, 1 MgCl_2_, 5 EGTA, 5 MgATP and 10 HEPES (titrated to pH 7.2 with KOH) and extracellular Tyrode solution comprised of (in mmol/L): 140 NaCl, 4 KCl, 2.5 CaCl_2_, 1 MgCl_2_, 10 glucose, and 5 HEPES (titrated to pH 7.4 with NaOH). Patch pipettes (A-M Systems Inc., USA, Schott #8250) were pulled on a dual stage glass micropipette puller (Narishige PC-10) and heat-polished (Narishige, MF 83) to obtain a final resistance of 2–3 MΩ. The cells on coverslips were transferred to a bath chamber mounted on the stage of an inverted microscope and continually superfused with Tyrode solution preheated to 37°C. Whole cell patch clamp recordings of I_HCN4_ were made using: 1) an Axopatch 1D amplifier (Axon Instruments) and a CV-4 headstage, with a Digidata 1200B or 1440A A-D interface (Molecular Devices) and Clampex software 10.7 (Molecular Devices); or 2) an Axopatch 200B amplifier (Axon instruments), digitization using an ITC-18 computer interface (Instrutech corporation) and HEKA Pulse software. Series resistance was compensated by 70%–75% (typically 2.5–7.5 MΩ). The I_HCN4_ currents recorded were filtered at 1–2 kHz and digitized at 10 kHz. Data acquired were analysed primarily using Clampfit 10.7 (Molecular Devices), Microsoft Excel 2204, and OriginPro 2021b. Cells with leak current of more than 100 pA at the −40 mV holding potential were not included in the analysis.

### Sequence alignments

Sequences were acquired from the National Centre for Biotechnology Information (NCBI) GenBank (accessed June 2020). Only HCN4 ortholog sequences with the complete whole channel sequence were selected and partial sequences generating an incomplete transcript were not included in the phylogenetic analysis. To minimise misalignment in the analysis, the full-length sequences were trimmed to amino acids 1 to 300 which included the full N-terminus and the start of the S1 domain which exhibits high sequence identity within the sequences used. Sequence alignments of the trimmed sequences and phylogenetic trees were generated using Clustal omega. Sequences that displayed little homology were removed from the alignment.

### Statistical analysis

Statistical analysis was performed using GraphPad Prism software. All quantitative data are presented as mean ± standard error of the mean (SEM). The statistical significance difference between the experimental groups were analysed using one-way analysis of variance (ANOVA) with Tukey’s *post hoc* multiple comparison tests. A Welch’s ANOVA was used to compare experimental groups with unequal standard deviations. Differences between experimental groups with *p*-values < 0.05 were considered statistically significant and accordingly denoted by * *p* < 0.05, ** *p* < 0.01, *** *p* < 0.001 and **** *p* < 0.0001.

## Results

### HCN4 is palmitoylated in multiple experimental model systems

**FIGURE 1:**
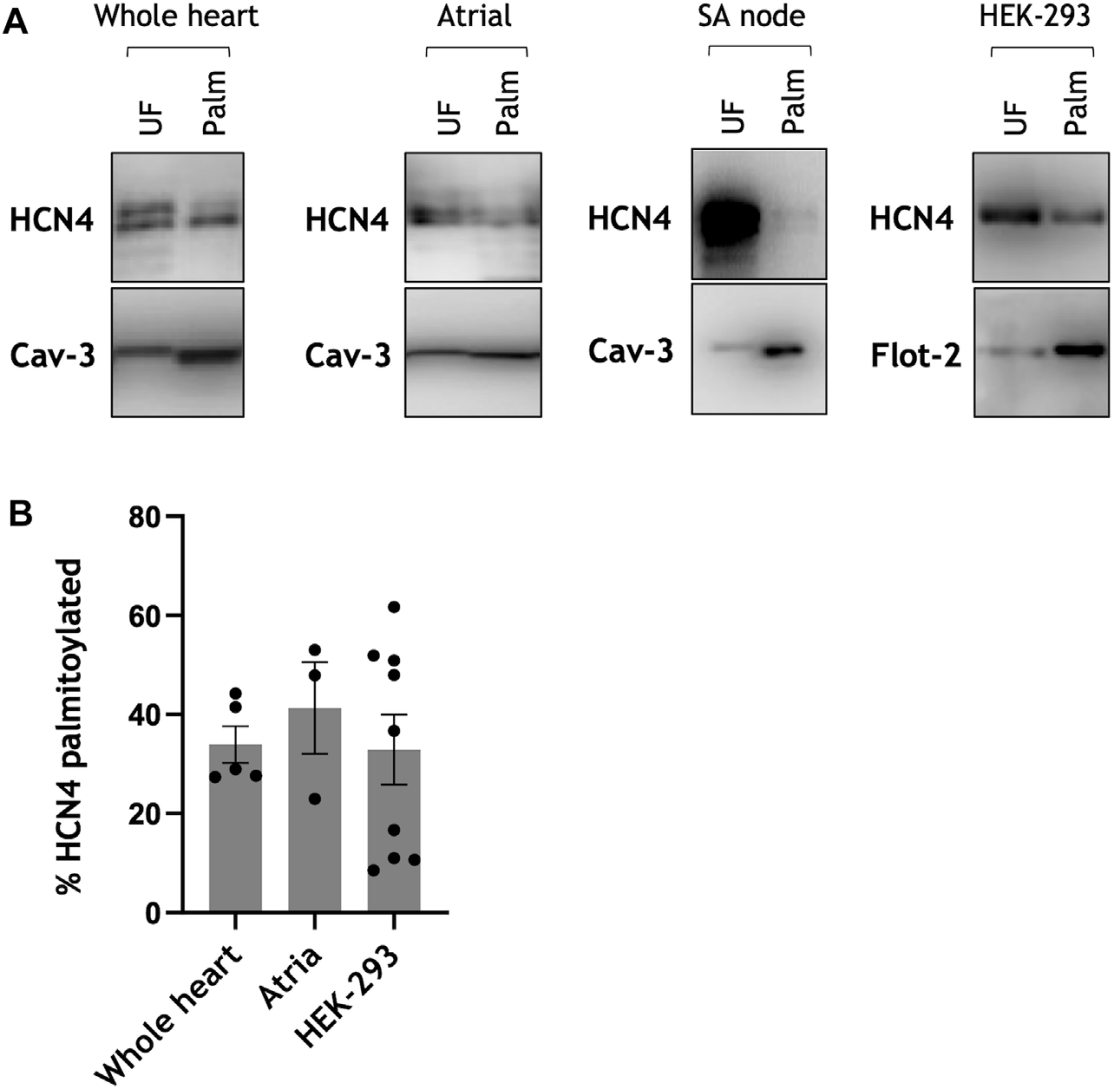
HCN4 palmitoylation in cardiac tissue and transfected cells. **(A)** Palmitoylation of HCN4 assessed using acyl-RAC from isolated rat neonatal whole heart, rat neonatal atria, rabbit sinoatrial node and following transient transfection of HEK-293 cells. The constitutively palmitoylated proteins caveolin-3 (Cav-3) and flotillin-2 (Flot-2) were used as a marker of assay efficiency. Full length HCN4 was detected with anti-HCN4 antibody. **(B)** Palmitoylation stoichiometry of endogenous HCN4 in rat neonatal cardiac tissue (whole heart, atria) and transiently transfected cells relative to caveolin-3 and flotillin-2, respectively. Compared to caveolin-3 the fraction of HCN4 palmitoylated is ∼33 ± 4% in rat neonatal whole heart (*n* = 5) and ∼41 ± 9% in rat neonatal atria (*n* = 3). Compared to flotillin-2 the fraction of HCN4 palmitoylated in HEK-293 cells is ∼33 ± 7% (*n* = 9). Each spot in the bar chart represents a measurement made from an independent population of cells. UF: Unfractionated cell lysate; Palm: palmitoylated protein.

We investigated palmitoylation of HCN4 using resin-assisted capture of acylated proteins (acyl-RAC), which purifies acylated proteins (but not those interacting with them) under strongly denaturing conditions. All assays were analysed by immunoblotting for HCN4 and a constitutively palmitoylated protein: caveolin-3 from muscle, and flotillin-2 from cultured cells (Figure 1A). HCN4 was palmitoylated in all systems investigated (whole neonatal rat hearts, neonatal rat atria, rabbit sinoatrial node, HEK-293 cells transiently transfected with HCN4). We estimated the fraction of HCN4 that is palmitoylated by comparing its enrichment in the acyl-RAC assay with the enrichment of the constitutively palmitoylated control proteins. Consistently 33%–40% of HCN4 was captured in these assays (Figure 1B), suggesting that two different populations of HCN4 are present in cardiac tissue: approximately one third of total HCN4 is palmitoylated and approximately two thirds is not palmitoylated.

### HCN4 palmitoylation sites are located in the protein’s amino terminus

**FIGURE 2:**
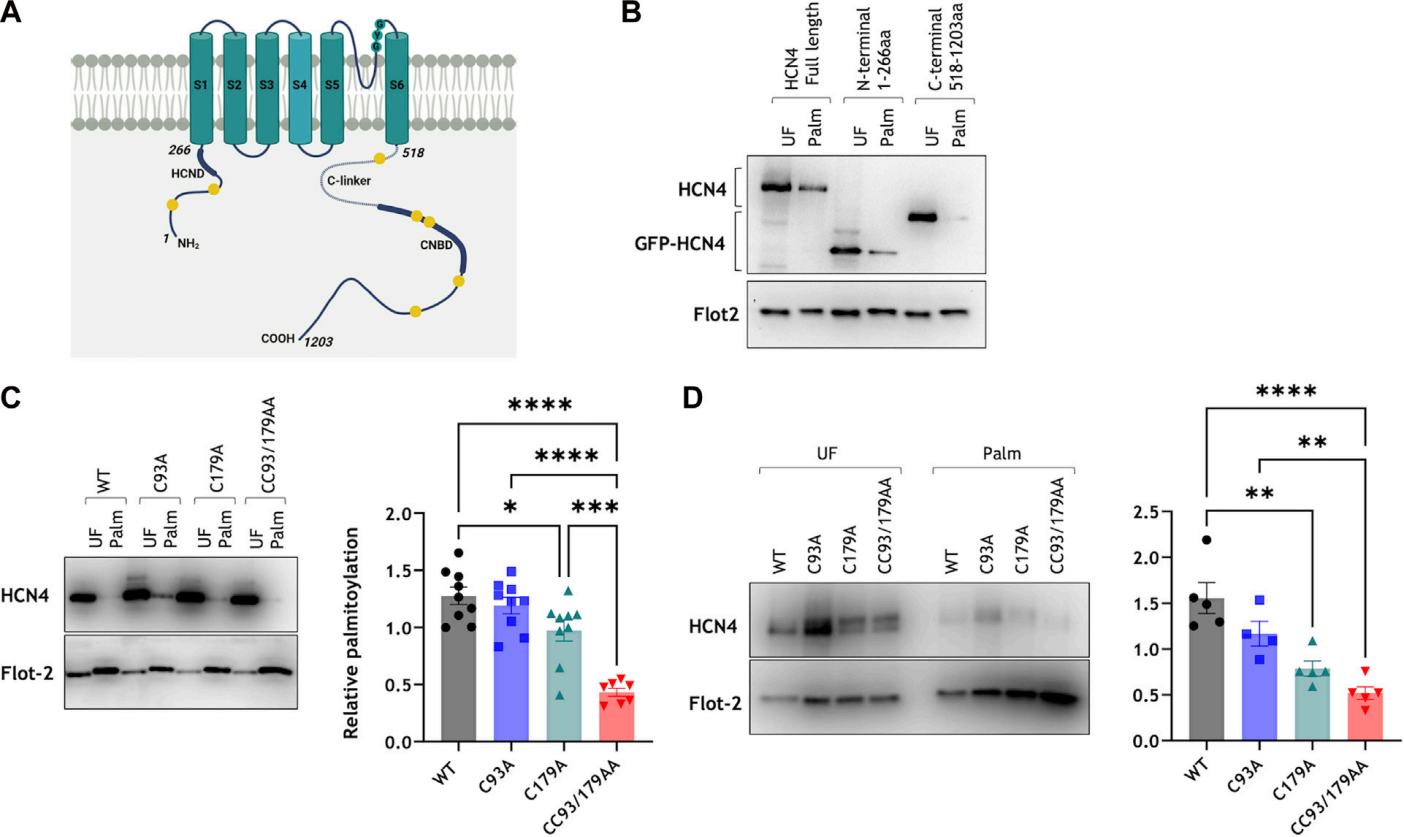
Palmitoylation site mapping in HCN4. **(A)** HCN4 schematic indicating the positions of the HCN domain (HCND) in the intracellular amino terminus, the C-linker and cyclic nucleotide binding domain (CNBD) in the intracellular carboxyl terminus, and all intracellular cysteines (yellow circles) in the amino (C93, C179) and carboxyl (C586, C662, C679, C755, C887) termini. **(B)** Palmitoylation of full length HCN4, YFP-N-terminus (1-266) and YFP-C-terminus (518-1203) in transiently transfected HEK-293 cells was assessed by acyl-RAC. Full length HCN4 was detected with anti-HCN4 antibody and the YFP fused N-/C- terminal fragments were detected with anti-GFP antibody. Flotillin-2 enrichment (Flot 2) confirms the efficiency of the acyl-RAC assay. UF, Unfractionated cell lysate; Palm: Palmitoylated proteins; representative figure of *n* = 8. **(C)** Site-directed mutagenesis on the YFP-fused N-terminus reveals cysteine 93 and 179 as palmitoylation sites. **(D)** Palmitoylation of stably expressed wild type and mutant HCN4 confirm cysteine 179 as the primary palmitoylation site of HCN4. * *p* < 0.05, ** *p* < 0.01, *** *p* < 0.001 and **** *p* < 0.0001, one way ANOVA followed by Tukey’s *post hoc* test. All groups are not significantly different from each other except where indicated.

We next set out to identify the palmitoylated cysteines in HCN4. Human HCN4 has seven intracellular cysteine residues: two in the amino terminus before the first transmembrane domain and five in the carboxyl tail after the sixth transmembrane domain (Figure 2A). In order to determine which region(s) of HCN4 contained the palmitoylation site(s) we fused the HCN4 amino terminus (1-266) or carboxyl terminus (518-1203) to YFP, expressed fusion proteins in HEK-293 cells and measured their palmitoylation by acyl-RAC (Figure 2B). Palmitoylation of the carboxyl terminal fusion protein was essentially undetectable, but a proportion of the amino terminal fusion protein was palmitoylated, suggesting one or both of the amino terminal cysteines are palmitoylated. We mutated one (C93A, C179A) or both (CC93/179AA) cysteines in the YFP fusion protein and assessed the palmitoylation status of the fusion proteins using acyl-RAC. Mutagenesis of C93 did not reduce the amount of YFP-tagged amino terminus captured by acyl-RAC, but mutagenesis of C179 did (Figure 2C). The modest reduction in the amount of C179A captured by acyl-RAC was significantly further reduced by mutagenesis of C93. The acyl-RAC assay captures proteins regardless of the number of palmitoylation sites occupied, and consequently does not differentiate between singly and doubly palmitoylated proteins. When a protein has one ‘dominant’ palmitoylation site and one ‘secondary’ palmitoylation site, if the secondary palmitoylation site is mutated then acyl-RAC still captures the protein *via* the dominant site. It is only when the dominant site has already been removed that the impact of mutating the secondary site can be seen. We conclude from these experiments that both cysteines in the fusion protein between YFP and the HCN4 amino terminus are palmitoylated, and that C179 may be the principal palmitoylation site.

To confirm the identity of the HCN4 palmitoylation sites in the full-length protein we generated 293T cells stably expressing tetracycline inducible wild type (WT), C93A, C179A and CC93/179AA HCN4. This system enables consistent expression levels in all cells investigated so is, in principle, considerably superior to transient transfection. Mutagenesis of C179 caused the greatest reduction in the amount of HCN4 captured using acyl-RAC, and mutagenesis of both amino terminal cysteines further reduced the amount of palmitoylated HCN4 (Figure 2D). A small amount (10% ± 2% of total HCN4) of CC93/179AA HCN4 was purified by acyl-RAC, so we do not rule out residual palmitoylation of cysteines in the HCN4 C terminus. However, since the most quantitatively significant palmitoylation occurred in the HCN4 N terminus, in subsequent experiments we focussed on identifying the functional consequences of palmitoylation in this region of HCN4.

### Palmitoylation does not influence cell surface delivery of HCN4

**FIGURE 3:**
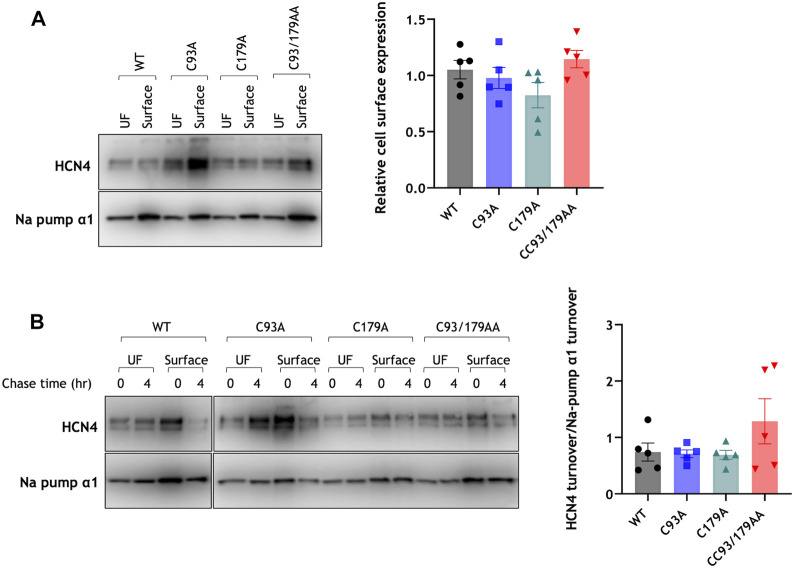
Palmitoylation and HCN4 trafficking. **(A)** Surface membrane proteins were prepared from stably transfected 293T cells using membrane-impermeable biotinylation reagents and immunoblotted alongside their corresponding unfractionated cell lysate. The plasma membrane associated housekeeper Na^+^/K^+^ ATPase α1 subunit (Na pump α1) was used as a marker of assay efficiency. The bar chart presents quantification of biotinylated HCN4 and Na^+^/K^+^ ATPase α1 subunit relative to its total expression and normalised to the daily experimental average. WT, wild type; UF, unfractionated cell lysate; *n* = 5. **(B)** Surface membrane HCN4 (surface) purified at 0 h and 4 h after biotinylation of surface membrane proteins were immunoblotted alongside their unfractionated cell lysate (UF). The plasma membrane associated housekeeper Na^+^/K^+^ ATPase α1 subunit (Na pump α1) was used as a marker of assay efficiency. HCN4 turnover was normalised to the turnover of housekeeper Na^+^/K^+^ ATPase α1 subunit. WT, wild type; UF, unfractionated cell lysate; *n* = 5. No groups are significantly different from each other.

Palmitoylation can regulate both anterograde transport of proteins through the secretory pathway to the plasma membrane (Ernst et al., 2018), and the rate of internalisation of proteins from the plasma membrane (Tulloch et al., 2011). We first investigated steady state expression of HCN4 at the surface membrane using membrane-impermeable biotinylation reagents. The quantity of HCN4 present at the cell surface was not different between cells expressing WT, C93A, C179A or CC93/179AA HCN4 (Figure 3A). Next, we assessed the rate of degradation of surface-localised HCN4 using pulse chase experiments. Surface membrane proteins were biotinylated and either prepared immediately or after a 4 h ‘chase’ (Figure 3B). We compared the fraction of biotinylated HCN4 remaining after the 4 h chase with the fraction of the housekeeping protein Na^+^/K^+^ ATPase α1 subunit in the same samples (Figure 3B). The relative rate of degradation of HCN4 was not different between the different cell lines, leading to a conclusion that palmitoylation does not influence trafficking or degradation of HCN4.

### Palmitoylation modifies the magnitude but not the activation properties of HCN4-mediated currents

**FIGURE 4:**
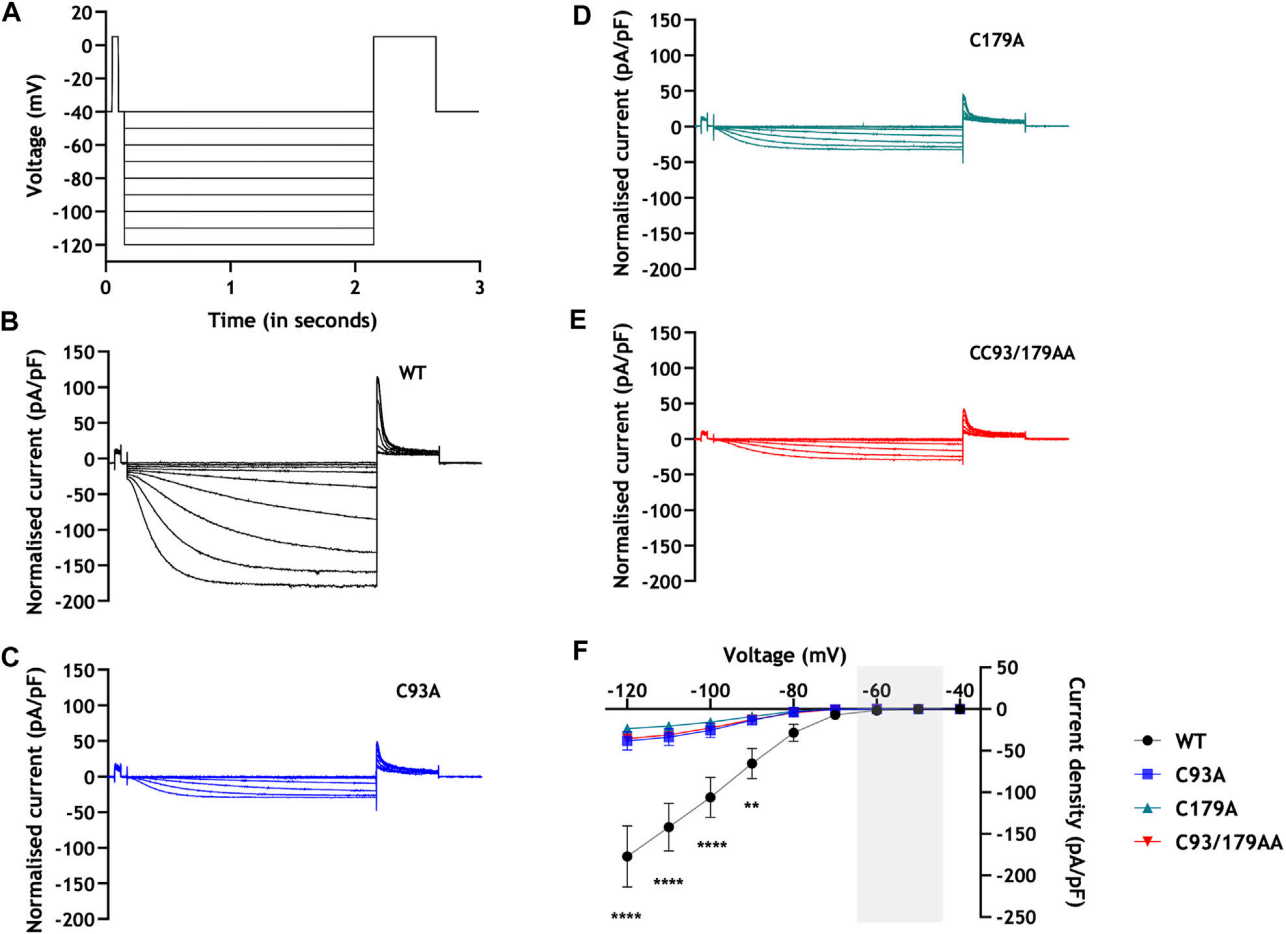
Palmitoylation and HCN4 currents: current-voltage relationships. **(A)** Voltage protocol. Two second hyperpolarising voltage steps were made from a holding potential of −40 mV. B-E: Representative families of currents from cells stably expressing WT **(B)**, C93A **(C)**, C179A **(D)** and CC93/179AA **(E)** HCN4. **(F)** The average steady-state current-voltage relationship from 293T cells stably expressing WT (black), C93A (blue), C179A (green) and CC93/179AA (red) HCN4. The sinoatrial diastolic depolarisation voltage range is highlighted in grey. **, *p* < 0.01; ****, *p* < 0.0001 (two-way ANOVA followed by Tukey’s *post hoc* comparison); *n* = 8-13.

**FIGURE 5:**
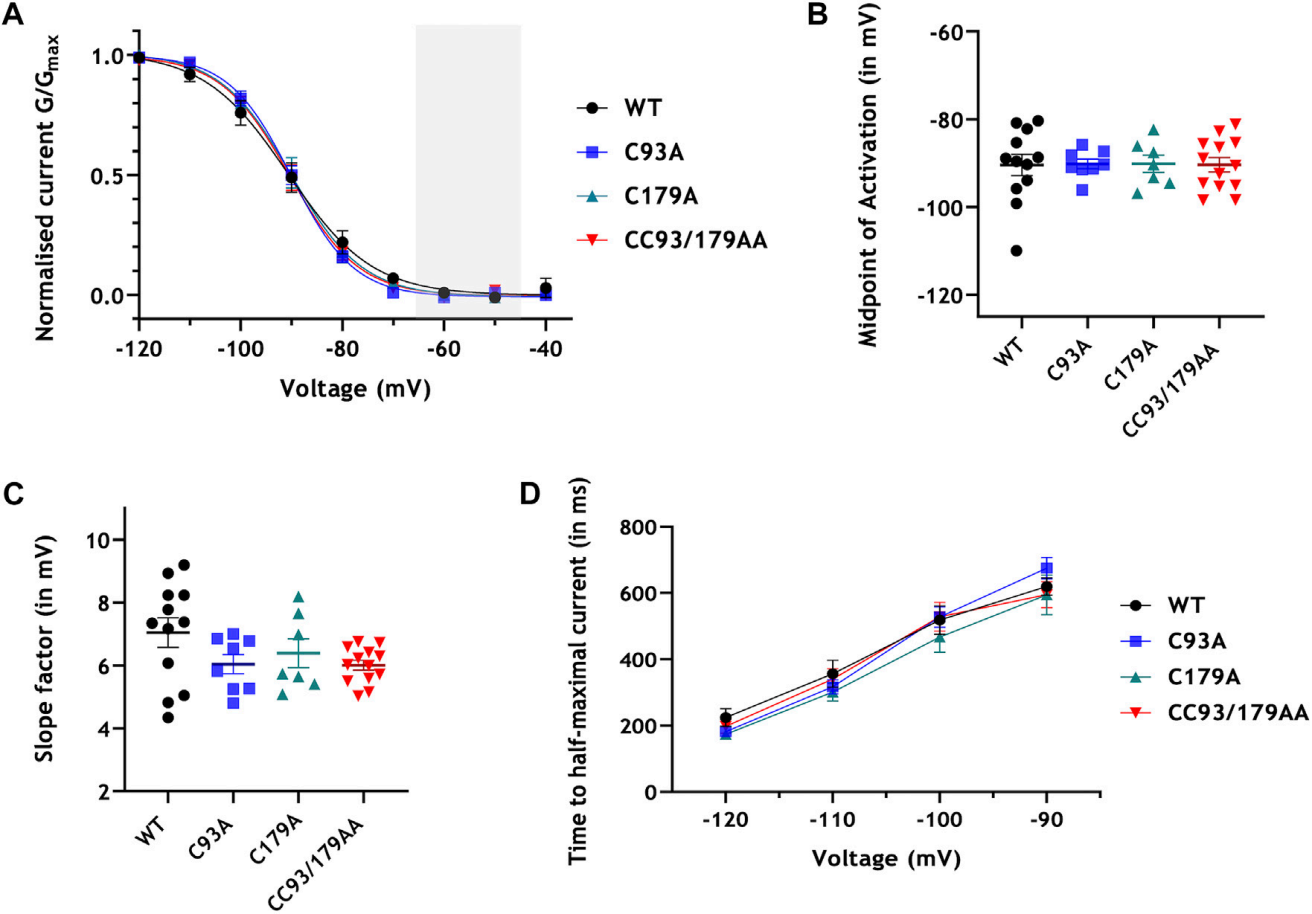
Palmitoylation and HCN4 currents: analysis. **(A)** Whole cell channel conductance (G) was normalised to the maximum conductance (G_max_) of each cell and fitted with a Boltzmann function. The sinoatrial diastolic depolarisation voltage range is highlighted in grey. **(B)** Half-maximal activation potentials (V_0.5_) obtained from the Boltzmann fits of the activation curves for each cell. **(C)** Slope factor (k) obtained from the Boltzmann fits of the activation curves for each cell. **(D)** Time to half-maximal activation at voltages −90 mV to −120 mV. No groups are significantly different from each other; *n* = 8-13.

We used whole-cell patch clamping to evaluate HCN4 current (I_HCN4_) in stably transfected 293T cells in which HCN4 expression was induced with tetracycline. Endogenous HCN4 is not expressed in HEK293 cells, but this expression system is well-validated to interrogate I_HCN4_ (Varghese et al., 2006). Representative currents and current voltage relationships are presented in Figure 4, and analysis of these currents is presented in Figure 5. In cells expressing WT HCN4, hyperpolarising voltage steps for 2 s from a holding potential of −40 mV (protocol shown in Figure 4A) resulted in a brief instantaneous current followed by a progressively developing sigmoidal time-dependent current characteristic of I_HCN4_. Depolarising to 5 mV for 0.5 s following the hyperpolarising test pulse elicited outward tail currents which inactivated in a voltage-dependent manner. These WT currents are similar to those reported in prior studies of HCN4 expressed in HEK cells (Milanesi et al., 2006; Nof et al., 2007; Schweizer et al., 2010; Liao et al., 2012). The peak amplitude of the current elicited by each of the hyperpolarising test pulses was measured and normalised to the corresponding whole cell capacitance and data pooled across cells (Figure 4F). The amplitude of currents conducted by WT HCN4 was significantly larger than that of palmitoylation site mutants in the voltage range −90 mV to −120 mV. The normalised whole cell conductance (G/Gmax) values were plotted against their corresponding test voltage and the resulting data fitted with a standard Boltzmann function in order to establish the voltage dependence of activation of WT and mutant HCN4 (Figure 5A). There was no significant difference in the half maximal activation voltage (V_0.5_) between the WT and mutant channels (Figure 5B: −90.2 ± 1.1 mV for C93A, −90.1 ± 2.0 mV for C179A, −90.4 ± 1.6 mV for CC93/179AA vs. −90.4 ± 2.5 mV for WT). Similarly, there were no significant changes in the slope factor for the activation relation between the WT and the mutant HCN4 channels (Figure 5C: 7.1 ± 0.5 mV for WT, 6.0 ± 0.3 mV for C93A, 6.4 ± 0.5 mV for C179A, 6.0 ± 0.2 mV for CC93/179AA). The time dependence of I_HCN4_ activation was quantified by measuring the time to half-maximal current over a range of the test potentials from −90 mV to −120 mV, for each cell. There was no difference between the activation time-course of WT and mutant channels (Figure 5D). We therefore conclude that palmitoylation significantly influences I_HCN4_ magnitude but not activation gating.

### Conservation of HCN4 palmitoylation sites

**FIGURE 6:**
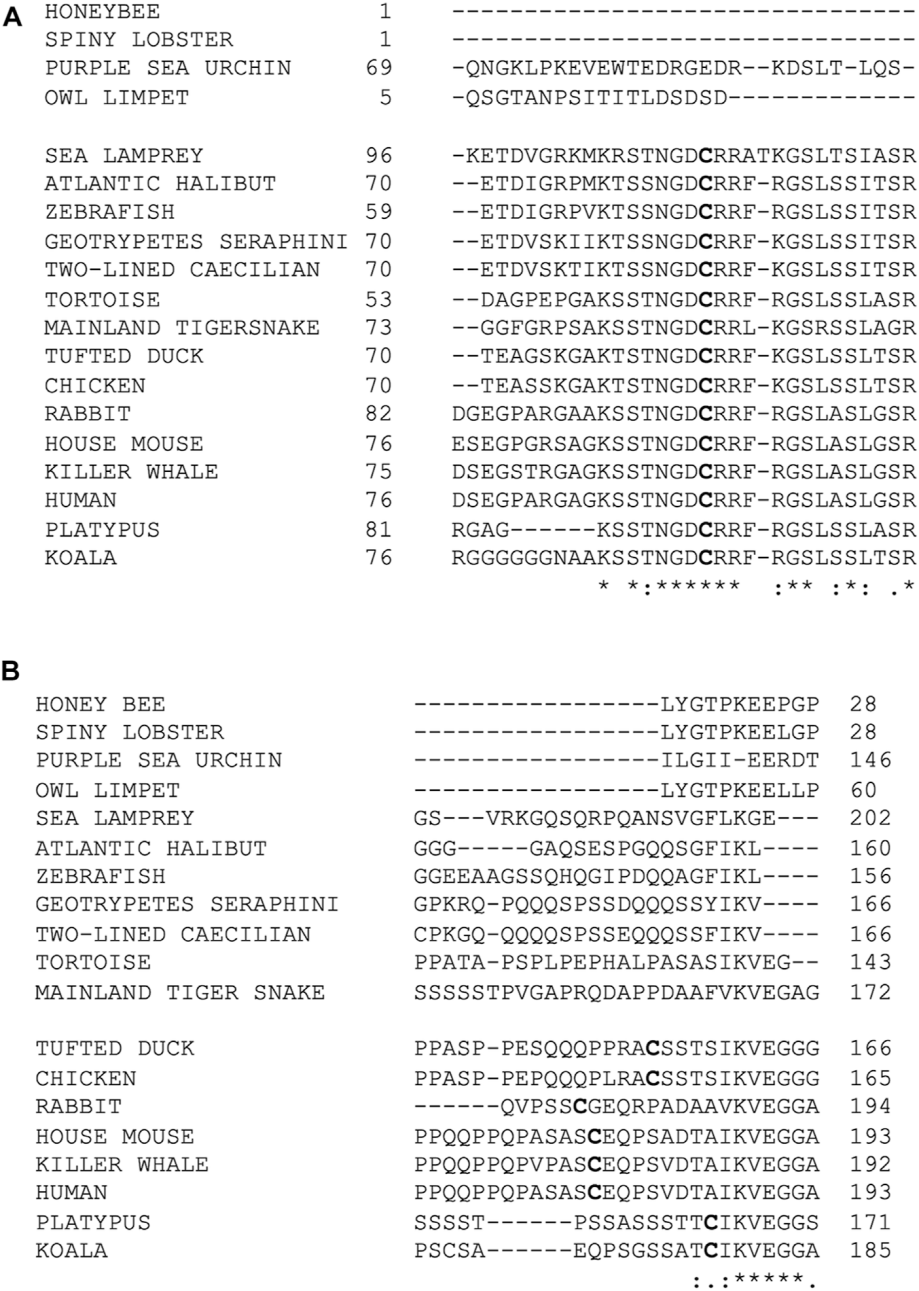
Conservation of palmitoylation sites amongst HCN4 homologues. **(A)** Conservation of cysteine 93. Alignment was generated using Clustal Omega. Palmitoylation site C93 and analogous cysteines are highlighted in bold. C93 is conserved in all vertebrates. The sequence surrounding C93 includes the cassette *SSTNGDCRRFKGSLSSLTSR* which is uniquely conserved amongst the vertebrates. **(B)** Conservation of cysteine 179. Alignment was generated using Clustal Omega. Palmitoylation site C179 and analogous cysteines are highlighted in bold. C179 is conserved in mammals and birds only.“*” below an amino acid indicates 100% conservation between isoforms; “:” indicates amino acids of highly similar properties; “.” indicates amino acids of weakly similar properties.

We investigated conservation of the HCN4 palmitoylation sites between species. A cysteine analogous to human C93 is present in all vertebrate HCN4 homologues but is absent from all invertebrates. In contrast, C179 is absent from invertebrates, fish, amphibia and reptiles but is conserved in mammals and possibly birds (Figure 6). These observations suggest that HCN4 regulation by palmitoylation is an adaptation specific for vertebrates and endotherms.

## Discussion

This study set out to investigate the functional impact of HCN4 palmitoylation. We identify two cysteines in the HCN4 amino terminus that are palmitoylated in mammalian cells. Mutation of one or both cysteines significantly reduces HCN4 palmitoylation and profoundly reduces the magnitude of HCN4-mediated currents. We therefore conclude that palmitoylation is a potent positive regulator of I_HCN4_. Given the importance of the funny current for diastolic depolarisation, HCN4 palmitoylation has the potential to exert a powerful influence on heart rate.

### Palmitoylation and I_HCN4_

The results of prior experiments on HCN1 and HCN2 channels have suggested that amino terminal interactions are important for HCN subunit assembly (Proenza et al., 2002). Combined deletion of the amino and carboxyl termini of HCN4 has been reported to result in a ∼10 mV hyperpolarizing shift in voltage dependence of current activation and to slow activation timecourse (Ishii et al., 2001). Comparison of brain and heart HCN4 variants with distinct amino termini, together with mutagenesis studies have also demonstrated a role for the amino terminus in HCN4 channel activation (Liu and Aldrich, 2011). Our results showed no significant difference in the activation parameters investigated between WT and unpalmitoylatable HCN4, but found a profound effect on the magnitude of I_HCN4_. Similar results to those shown here for inducible HCN4 expression (Figures 4, 5) were seen in additional experiments (data not shown) using transient transfection. Macroscopic current magnitudes through any voltage sensitive channel are determined by the number of channels present at the cell surface (*N*), the single channel conductance (*G*), and the channel open probability (*Po*). We found no obvious influence of palmitoylation in the HCN4 amino terminus on steady state abundance of HCN4 protein at the cell surface membrane. We hence rule out any significant influence of palmitoylation on *N*. Since the HCN4 amino terminus is relatively far from the channel’s pore (Saponaro et al., 2021), it seems unlikely that palmitoylation produces a profound alteration in *G*. We therefore propose that HCN4 *Po* is regulated by palmitoylation. There is precedent for the single channel properties and *Po* of HCN4 to be regulated by the presence of an accessory subunit (Brandt et al., 2009). It is also well-established that HCN4 gating is not adequately described by a second-order Hodgkin-Huxley model (DiFrancesco et al., 1986; Hoppe et al., 1998). Five closed and five open states have been proposed (Altomare et al., 2001), and experimental recordings of single channel events are consistent with the existence of numerous open and closed states (Michels et al., 2005). Clearly there is considerable scope for post-translational events to control the lifetime of one or more state and consequently regulate HCN4 *Po*. However, we do not rule out the possibility that ‘silent’ channels in the plasma membrane are recruited by palmitoylation. Co-operative gating of individual HCN2 channels has also been described (Dekker and Yellen, 2006). If co-operativity were enhanced by HCN4 palmitoylation this would manifest as increased *Po* and larger currents. Future experiments measuring single channel activity in WT and unpalmitoylated HCN4 mutants are required to distinguish these possibilities. Experiments to determine how cAMP regulates palmitoylated and non-palmitoylated HCN4 are also a high future priority. Although cAMP binds to the channel’s carboxyl terminus we do not rule out the possibility that it differentially regulates channels based on the palmitoylation status of their amino terminus.

The typical consequence of protein palmitoylation is for the palmitoylated region of a protein to be anchored to the membrane (Main and Fuller, 2022). In the case of HCN4 our results are consistent with ‘pinning’ of the channel’s amino terminus to the membrane leading to enhanced *Po*. This implies either that in the unpalmitoylated state the HCN4 amino terminus (which is predicted to be highly disordered) negatively regulates channel activity or that in the palmitoylated state the amino terminus is a channel facilitator.

AlphaFold predicts the amino terminus of HCN4 to be extensively disordered, and no secondary structure was observed in this region of the protein in a recent HCN4 cryoEM structure (Saponaro et al., 2021). We acknowledge the possibility that cysteine to alanine mutagenesis of the HCN4 amino terminus may alter HCN4 behaviour independent of changes in palmitoylation. However, given the lack of structure in this region of the protein we suggest that the conservative substitutions introduced in this investigation are unlikely to significantly impact HCN4 folding.

### Palmitoylation and HCN4 oligomerisation

We observe a substantial functional impact of palmitoylation on I_HCN4_ when only ∼33% of HCN4 is palmitoylated. Mature HCN4 channels are tetrameric (Saponaro et al., 2021). If palmitoylation has no influence on how monomers assemble to form this tetramer, then the probability of a tetramer assembling from entirely non-palmitoylated HCN4 is 0.67^4^: approximately 0.2. Hence, we predict approximately 80% of HCN4 tetramers will contain one or more palmitoylated subunits. The oligomerisation of individual subunits offers an elegantly efficient way to achieve substantial functional differences even at a relatively low overall palmitoylation stoichiometry. If palmitoylation doesn't influence oligomerisation then mature HCN4 formed from a single palmitoylated subunit would be the most common form, but a significant proportion formed from two palmitoylated protomers would also exist. Future experiments using gain of function approaches to enhance HCN4 palmitoylation [for example, (Li et al., 2020)] will need to address whether there are functional differences between mature HCN4 formed from single or multiple palmitoylated monomers.

### Palmitoylation and the regulation of other HCN channels

Although the present study is the first detailed study of sites and consequences of palmitoylation of HCN4, palmitoylation of HCN1, 2 and 4 has been described by others (Itoh et al., 2016). Multiple cysteines in the HCN2 amino terminus are palmitoylated, but no functional effect was observed when these sites were mutated. However, this earlier investigation relied on biochemical measurements of palmitoylation only in HEK cells but functional experiments only in *Xenopus* oocytes, and did not present evidence that HCN2 is palmitoylated in oocytes (Itoh et al., 2016). The lack of conservation of the human HCN4 palmitoylation sites in amphibians means it is doubtful whether oocytes possess the necessary enzymatic machinery to palmitoylate HCN channels. In addition, the membrane properties of an oocyte at room temperature are very different to a mammalian cell at 37°C. Since palmitoylation typically alters the relationship between an integral membrane protein and the phospholipid bilayer in which it resides, the usefulness of the oocyte system to evaluate the functional impact of palmitoylation is questionable. We suggest that the possibility that palmitoylation regulates important functional properties of other HCN channels in mammalian expression systems is one worthy of further pursuit.

### Palmitoylation sites in HCN4

In our experiments, mutagenesis of the HCN4 amino terminal cysteines significantly reduced, but did not abolish its palmitoylation. We consider the small amount of residual CC93/179AA HCN4 captured in our palmitoylation assays to be largely ‘background’, but we do not rule out the possibility of a low level of palmitoylation in the channel’s C terminus. The HCN4 CryoEM structure does not resolve large regions of the amino (1-214) and carboxyl (716-1023) termini. Those cysteines in the C terminus that are resolved (C586, C662, C679) are not positioned near membrane, making them unlikely palmitoylation sites [juxtamembrane cysteines are typically palmitoylated because the active site of the palmitoylating enzymes lies at the membrane/cytosol interface (Rana et al., 2018)]. We cannot rule out palmitoylation of C755 or C887.

## Conclusion and future directions

The data in this study collectively indicate that native HCN4 protein in cardiac tissue is palmitoylated and reveal the importance of palmitoylation sites in the amino terminus of HCN4 channels. Further work, likely including measurements at the single channel level, is required to elucidate the mechanism(s) by which the loss of palmitoylation reduces I_HCN4_ magnitude. The differences in I_HCN4_ amplitudes observed here were significant at relatively negative membrane voltages. In order to determine the consequences of the loss of HCN4 amino terminal palmitoylation on the channel activity over diastolic depolarization voltages and function of the intact SAN, the future use of appropriately genetically modified mice is now warranted. This would allow effects of HCN4 modification by palmitoylation to be ascertained for the intact native SAN in the absence of whole cell dialysis. The present study provides a strong foundation from which such work can be undertaken.

## Data Availability

The raw data supporting the conclusion of this article will be made available, upon request, without undue qualification.
